# A Research Agenda for Helminth Diseases of Humans: Modelling for Control and Elimination

**DOI:** 10.1371/journal.pntd.0001548

**Published:** 2012-04-24

**Authors:** María-Gloria Basáñez, James S. McCarthy, Michael D. French, Guo-Jing Yang, Martin Walker, Manoj Gambhir, Roger K. Prichard, Thomas S. Churcher

**Affiliations:** 1 Department of Infectious Disease Epidemiology, School of Public Health, Faculty of Medicine (St Mary's campus), Imperial College London, London, United Kingdom; 2 Queensland Institute of Medical Research, University of Queensland, Herston, Australia; 3 Schistosomiasis Control Initiative, Department of Infectious Disease Epidemiology, School of Public Health, Faculty of Medicine (St Mary's campus), Imperial College London, London, United Kingdom; 4 Department of Schistosomiasis Control, Jiangsu Institute of Parasitic Diseases, Jiangsu, People's Republic of China; 5 MRC Centre for Outbreak Analysis and Modelling, Department of Infectious Disease Epidemiology, School of Public Health, Faculty of Medicine (St Mary's campus), Imperial College London, London, United Kingdom; 6 Institute of Parasitology, McGill University, Montreal, Canada; National Institute of Parasitic Diseases China CDC, China

## Abstract

Mathematical modelling of helminth infections has the potential to inform policy and guide research for the control and elimination of human helminthiases. However, this potential, unlike in other parasitic and infectious diseases, has yet to be realised. To place contemporary efforts in a historical context, a summary of the development of mathematical models for helminthiases is presented. These efforts are discussed according to the role that models can play in furthering our understanding of parasite population biology and transmission dynamics, and the effect on such dynamics of control interventions, as well as in enabling estimation of directly unobservable parameters, exploration of transmission breakpoints, and investigation of evolutionary outcomes of control. The Disease Reference Group on Helminth Infections (DRG4), established in 2009 by the Special Programme for Research and Training in Tropical Diseases (TDR), was given the mandate to review helminthiases research and identify research priorities and gaps. A research and development agenda for helminthiasis modelling is proposed based on identified gaps that need to be addressed for models to become useful decision tools that can support research and control operations effectively. This agenda includes the use of models to estimate the impact of large-scale interventions on infection incidence; the design of sampling protocols for the monitoring and evaluation of integrated control programmes; the modelling of co-infections; the investigation of the dynamical relationship between infection and morbidity indicators; the improvement of analytical methods for the quantification of anthelmintic efficacy and resistance; the determination of programme endpoints; the linking of dynamical helminth models with helminth geostatistical mapping; and the investigation of the impact of climate change on human helminthiases. It is concluded that modelling should be embedded in helminth research, and in the planning, evaluation, and surveillance of interventions from the outset. Modellers should be essential members of interdisciplinary teams, propitiating a continuous dialogue with end users and stakeholders to reflect public health needs in the terrain, discuss the scope and limitations of models, and update biological assumptions and model outputs regularly. It is highlighted that to reach these goals, a collaborative framework must be developed for the collation, annotation, and sharing of databases from large-scale anthelmintic control programmes, and that helminth modellers should join efforts to tackle key questions in helminth epidemiology and control through the sharing of such databases, and by using diverse, yet complementary, modelling approaches.

## Introduction

It is generally accepted that mathematical models have an important role to play in our understanding of the processes underlying observed epidemiological patterns of the helminthic diseases that afflict humankind. Models have been shown to provide important insights into the mechanisms responsible for persistence, resilience, and stability of helminth infections. A key example is the dependence on parasite density, a concept foreign to most other infectious diseases [1]. However, in very few cases has the potential of models to provide critical insights to inform helminth research and practice at laboratory, clinical, epidemiological, operational, or policy levels been reached. An exception to this is the use of the microsimulation model ONCHOSIM by the Onchocerciasis Control Programme in West Africa (OCP) [2]. Notably absent has been the development of models to investigate or prepare for emerging/re-emerging infections and public health research, particularly in the context of the challenges posed by ambitious control and elimination programmes. This omission limits our ability to understand and predict population behaviour, under anthropogenic or natural change (including control interventions and climate change), of multi-host parasite systems, multi-parasitised host populations, parasites with complex transmission routes, transmission involving various vectors or intermediate hosts, and the spread of strains resistant to interventions including insecticides, molluscicides, anthelmintic drugs, or vaccines.

The appreciation of the key contribution that helminth modelling can potentially make to research and policy for the control and elimination of helminth diseases of humans was recognised in the identification of mathematical modelling as one of the five umbrella priorities of the Disease Reference Group on Helminthiases (DRG4) [3], established by the Special Programme for Research and Training in Tropical Diseases (TDR). Among the objectives of the modelling group were those of reviewing the current status of mathematical models for helminth infections of humans, placing it in a historical and a contemporary perspective, identifying recent advances and research gaps in helminth modelling, defining priorities and time horizons for the closing of such gaps, and outlining a research and development agenda for modelling within a more comprehensive research agenda for the control and elimination of human helminthiases.

In this paper, salient historical developments in helminth infection modelling are outlined, a summary of current frameworks and their main features is presented, and key modelling priorities to aid the implementation, monitoring and evaluation (M&E), and surveillance of programmes for the control and elimination of the human helminth infections under the remit of the DRG4 are discussed, with a focus on the soil-transmitted helminthiases (STHs), intestinal and urinary schistosomiasis, the filariases (lymphatic filariasis [LF] and onchocerciasis), food-borne trematodiases, and taeniasis/cysticercosis. Although a discussion of models for helminthiases of veterinary importance is outside the scope of this paper, we direct the readers to the excellent resource of [4], and will refer to these models when they have informed thinking on helminthic diseases of humans (e.g., models for investigation of anthelmintic resistance). Box 1 lists the abbreviations used in this paper.

**Box 1. List of Abbreviations**

**ABR,** annual biting rate

**APOC,** African Programme for Onchocerciasis Control

**DRG4,** Disease Reference Group on Helminth Infections

**DtW,** Deworm the World

**GNNTD,** Global Network for Neglected Tropical Diseases

**GPELF,** Global Programme to Eliminate Lymphatic Filariasis

**LF,** lymphatic filariasis

**malERA,** Malaria Eradication Research Agenda

**MDA,** mass drug administration

**MIDAS,** Models of Infectious Disease Agent Study

**M&E,** monitoring and evaluation

**NTD,** neglected tropical disease

**OCP,** Onchocerciasis Control Programme in West Africa

**OEPA,** Onchocerciasis Elimination Program for the Americas

**PPC,** Partners for Parasite Control

***R*** **_0_,** basic reproduction ratio

***R*** **_E_,** effective reproduction ratio

**SCI,** Schistosomiasis Control Initiative

**STHs,** soil-transmitted helminthiases

**TBR,** threshold biting rate

**TDR,** Special Programme for Research and Training in Tropical Diseases

**UNICEF,** United Nations Children's Fund (formerly United Nations International Children's Emergency Fund)

**UNDP,** United Nations Development Programme

**WHO,** World Health Organization

## A Brief History of Helminth Models

**Figure 1:**
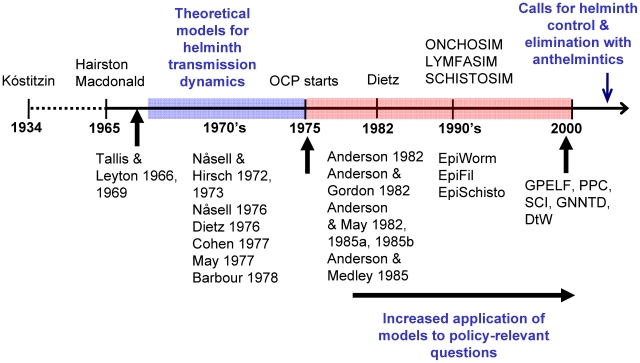
A historical timeline of mathematical models for helminthiases. Some of the pivotal papers that provided the foundation to the mathematical frameworks that are used for modelling helminth infections are highlighted (for a detailed explanation see main text; for a summary of current models see Table S1). Most of the work published until the 1980s (with the exception of papers by Hairston) largely consisted of theoretical frameworks that were motivated, but not fitted to epidemiological data. From that point onwards there has been an increased interest in parameterising models with data on the natural history of the infections, moving away from purely theoretical explorations. The deterministic and microsimulation models of the 1990s were strongly linked to the notion of providing decision support to control programmes (e.g., ONCHOSIM and the OCP in West Africa). Since the year 2000 there has been a steep increase in large-scale initiatives mostly reliant on anthelmintic drugs for the control and elimination of these parasitic infections, but this has not yet been accompanied by a comparable impetus towards using robust modelling to inform and guide such initiatives, though the GPELF has used LYMFASIM and EpiFil, and the SCI has used modified versions of EpiSchisto.

The first quantitative approach to the study of helminth infection is due to Kóstitzin [5], who in 1934 presented a formulation to describe the flow of hosts along a series of infection categories defined by increasing worm burden, introducing the all important notion of parasite density. Using surveys of parasite prevalence with age, Hairston in 1965 estimated rates of acquisition and loss of schistosomiasis by applying for the first time catalytic, force of infection models to the analysis of helminth infections [6]. During the same year, it was Hairston who first used snail and helminth life-tables to estimate the reproduction ratio of schistosomes at endemic equilibrium as well as the transmission probabilities between definitive and snail hosts from field data [7]. Also in 1965, Macdonald formulated mathematically the mating probability for a (randomly distributed) helminth species with separate (male and female) sexes (known as dioecious parasites), and introduced the concept of the transmission breakpoint [8] with reference to schistosomiasis. Whilst these formulations had been deterministic, Tallis and Leyton in 1966 [9] were the first to present stochastic models for the dynamics of dioecious parasites in their vertebrate hosts motivated by directly transmitted helminth species of veterinary importance; Leyton in particular formulated sexual mating functions in 1968 [10], and Tallis and Leyton introduced in 1969 the immigration-death framework [11]. During the 1970s there were important theoretical developments with a focus on schistosomiasis (Nåsell and Hirsch in 1972 and 1973 [12], [13]; Nåsell in 1976 [14], [15]; Cohen [16] and May [17] in 1977; Barbour in 1978 [18]), and on the vector-borne filarial nematodes (Dietz in 1976 [19]). In 1975, the commencement of the Onchocerciasis Control Programme in West Africa (OCP) would act as a catalyst for the use of epidemiological models in large-scale interventions [2], and in 1982 Dietz [20] presented deterministic and stochastic onchocerciasis models. From this time onwards there has been a great increase in the development of mathematical models for human helminthiases (see Table S1); thus, we focus here on some salient contributions, highlighting the models of Anderson and co-workers [21]–[25] in the 1980s, and the stochastic microsimulation approaches [1], [26]–[28] and their deterministic counterparts [29]–[31] of the 1990s. Since the year 2000 there has been an unprecedented global effort to control human parasitic infections at much larger geographical scales than previously, but it is our contention that this has not been accompanied by a proportionate increase in the influence that mathematical models could have in supporting such programmes. Figure 1 provides a schematic timeline of the development of helminth models.

## Current Status of Mathematical Models for Human Helminthiases

Although various mathematical models can be recognised in this context, including statistical models that have important roles in hypothesis testing and parameter estimation [32], the focus in this review is on population dynamics models. Parasite population dynamics models seek to describe the changes with respect to time (and host age where appropriate) of parasite abundance (infection prevalence and intensity) in humans and intermediate hosts or vectors at baseline (endemic equilibrium, prior to control) and during an intervention. They are based on our current understanding of the parasites' population biology and transmission dynamics, and describe how the life stages in the definitive host, environment, or intermediate hosts/vectors are inter-connected in the parasite's life cycle through contact, transmission, establishment, and parasite fecundity rates. Models can be deterministic or stochastic, population-based or individual-based, and may track infection intensity and/or infection prevalence (for a glossary of these approaches in helminth modelling, see [33] and Box 2 therein).

In this review, the role that mathematical models can play in various activities will be examined. These include: i) encapsulating current understanding of the population biology of the parasite, enabling study and description of the determinants of endemic (pre-control) equilibrium; ii) facilitating exploration of the impact on infection and morbidity of different control scenarios (e.g., single intervention strategies, modes of delivery, combinations of interventions); iii) estimating unknown or unobservable parameters by fitting models to data; iv) investigating the conditions for parasite elimination and the behaviour of the host–parasite system in the vicinity of transmission breakpoints; and v) exploring the evolutionary outcomes of control (e.g., spread of anthelmintic resistance). Table S1 presents a (non-exhaustive) summary of current mathematical models for helminth parasites, emphasising those with public health implications, and illustrates their use for each of these roles.

### Models for Helminth Population Biology

Helminth parasite population models explore the impact of various population and regulatory processes regarding the rates of parasite establishment, development, mating, fecundity, survival, and transmission, as well as the impact of overdispersed parasite distributions among definitive and vector hosts [1]. These models can be fitted to baseline data and can be used to contrast hypotheses about the generation and consequences of infection heterogeneity, and the operation of density-dependent (including immunologically mediated) processes, among others, by comparing statistically the resulting fits [34]. These models can be updated as new data and knowledge become available. The interactions between positive (facilitating) and negative (constraining) regulatory processes (Text S1), and of these with parasite distribution and anthelmintic treatment, have been investigated [24], [35], [36].

### Models for Exploration of Control Scenarios

Once the model has been developed and calibrated with appropriate parameter values (specific to the helminth–host system and location), it can be run until the endemic equilibrium steady state has been attained, after which interventions such as antiparasitic, antivectorial, snail control, and other measures [37] may be simulated. Among the antiparasitic measures that have been explored with models are the effects of chemotherapeutic treatment distributed either in the modality of mass drug administration (MDA), age-targeted (e.g., school-aged children), selective treatment (of given occupational groups) [24], [38], or vaccination [39], [40].

Assumptions regarding parasite life span and distribution of survival times, treatment efficacy, drug actions against various parasite life stages, coverage levels, and modalities of compliance can be incorporated and investigated [41]. Some models have also included acquired, protective immunity and looked at its interactive effect with control interventions [42], [43]. Model validation at this stage has been usually conducted by comparing how well model outputs reproduce observed data and resulting epidemiological trends during the intervention(s) under investigation [29]. Such model outputs may represent changes in worm burden, prevalence of infection and heavy infection, and changes in associated morbidity [44]. The latter itself requires careful investigation of disease outcomes related to infection, taking into account that co-infection with other parasites may be present, and that it is still unclear how present or past infection relates to measurable morbidity. However, a close fit between observed data and model outputs does not necessarily mean the model has captured the true underlying processes. The process of assessing structural validity, i.e., ascertaining that the model exhibits the right behaviour for the right reasons, is considered to be a more stringent measure of validation and has led to the devising of formal structural validity procedures. These include verification of structural assumptions (whether model structure is consistent with relevant and updated knowledge of the system being modelled) and parameter assumptions (model calibration), among others [45]. The literature on model validation is ample and controversial [46], as there are no fixed and universally agreed standards for selecting what test procedures or criteria to use for validation [47].

Stochastic models that incorporate parameter uncertainty can produce model outcomes ranging between upper and lower bounds, within which the data may be contained. Use and development of statistical methods for model fitting and comparison (both between alternative models, and between model outcomes and data) constitute an area of active research to be encouraged in the field of helminth mathematical models. This would strengthen our ability to understand and represent underlying processes and interpret modelling results. Although deterministic, population-based models may be suitable to investigate the average parasite population behaviour during the simulated control strategy, stochastic, individual-based models are more appropriate to investigate the probability of parasite elimination. However, Allee-type effects, introduced by facilitating density dependencies, allow elimination to occur in deterministic frameworks too [48].

### Models for Estimation of Directly Unmeasurable Parameters

With increasing use of advanced statistical methods (more recently including Bayesian approaches) that can now be implemented given the current availability of faster computing and more efficient algorithms, parasite epidemiology researchers are able to fit dynamic models to data for estimation of unknown parameters of interest. This has permitted estimation of parasite life span [49], [50]; treatment efficacy and drug effects on different parasite life stages [51], [52]; variation in host immune response to parasite life stages [53]; and transmission parameters such as parasite establishment rates [54], [55]. Such information is highly relevant but difficult to obtain by direct observation and/or experimentation. Recent examples in the field of schistosomiasis include measuring the transmission between hosts and snails in multi-host models of *Schistosoma japonicum* [56], and measuring the reductions in the force of infection (incidence of new infections) of *S. mansoni* resulting from large-scale implementation of praziquantel treatment [57]. However, there are also likely to be situations for which it is difficult to estimate separately highly correlated parameters, regardless of using the most advanced statistical methods. In these instances it may be necessary to either aggregate parameters in composite terms or seek data that may shed light on processes that are directly unmeasurable.

### Models for Helminth Elimination

Although the goal of some programmes is that of morbidity control and elimination of the public health burden of the diseases (STHs, schistosomiasis and, up until recently, onchocerciasis in Africa), others aim at interruption of transmission and eventual elimination of the parasite reservoir (LF, onchocerciasis through vector control/elimination in Africa, onchocerciasis in Latin America, dracunculiasis). The models to inform parasite elimination should generally be stochastic because as parasite density decreases, stochastic variations (and stochastic fade-out) will be more important than the mean behaviour. (This is partly due to demographic stochasticity; worms are individuals, not fractions.) As the simulated intervention programmes reach their end points, the parasite population will die out in some model runs but not in others. By undertaking many model runs, each model run with a different set of parameter values randomly chosen from a plausible range, statements can be made regarding the probability of parasite elimination that will result from a given intervention or combinations thereof [41], [58]. This would permit investigation of the influence of factors such as initial endemicity level, transmission intensity, host heterogeneity (exposure, susceptibility, predisposition), parasite overdispersion, vector or intermediate host competence and vectorial capacity (the latter including vector density and biting rate on humans), treatment frequency, duration and coverage required, synergistic effects of vector/snail control, etc.

Whereas programmes aimed at morbidity control may benefit from age-targeted chemotherapy of those hosts at higher risk of acquiring heavy infection and subject to the greatest morbidity, parasite elimination will require prolonged mass treatment of all infected individuals at all endemicity levels, and in all endemic communities if parasite eradication is the goal. (For definitions of parasite control, elimination, eradication, and extinction see [59].) Such elimination strategies will substantially shrink the size of susceptible parasite refugia (populations of untreated parasites, not subjected to drug pressure [60]), an important factor influencing the spread of anthelmintic resistance. Parasite elimination programmes that rely on chemotherapy alone must therefore put in place careful surveillance systems for prompt detection of transmission resurgence, suboptimal parasite clearance rates, and monitoring of drug efficacy (parasite susceptibility). The same applies for those programmes relying on vector/snail control because of the possibility of insecticide/molluscicide resistance.

### Transmission Thresholds and Breakpoints

Transmission breakpoints should not be confused with the threshold for transmission, known as the basic reproduction number or *R*_0_, or related parameters such as the threshold biting rate (TBR) in vector-borne infections [20]. Transmission breakpoints refer to finite parasite densities below which the parasite population would not be able to maintain itself; the basic reproduction number is, by definition, density independent. *R*_0_ represents the threshold condition for parasite invasion and persistence, as it has to be greater than 1 for the parasite population to reach its endemic state. It is possible to rearrange the equations of *R*_0_ for each helminth infection to derive threshold population sizes of definitive, intermediate, and vector hosts and demonstrate, for instance, that STHs can persist in human populations of much smaller sizes than those required for viral infections such as measles [61]. For the (vector-borne) filarial nematodes it is also possible to calculate TBR values below which the infection would not persist. These depend, in part, on the proportion of bites that vectors take on humans [20], [54], [62], emphasising the important role of measures that reduce vector density, measured by the annual biting rate (ABR), and vector-human contact. However, *R*_0_ is a somewhat idealised, parasite density–independent entity. In reality, many transmission processes depend on parasite density, so the quantity of interest becomes the effective reproduction ratio (*R*_E_), the composite parameter that reflects the changes in the transmission potential of a parasite with changes in parasite density [35], [63]. The value of *R*_E_ will be equal to 1 at endemic equilibrium (each female worm in the population replaces itself) and also at the so-called unstable equilibrium, the “elusive” transmission breakpoint or “holy grail” of parasite elimination. For dioecious parasites (those with separate sexes) and in those host–parasite systems with facilitating types of density dependence of the type described in Text S1, there will be unstable equilibrium parasite densities below which the parasite population would, in principle, become locally extinct (because females will not be mated and/or parasites will not establish within humans or vectors), and above which the parasite population will return to endemic equilibrium (Text S2 and Figure S1).

Understanding the behaviour of the host–parasite system in the vicinity of these transmission breakpoints is a priority area of research that requires the concourse of cross-disciplinary approaches such as mathematical analysis, knowledge of vector–host–parasite interactions, parasite population biology, and epidemiology. The values of helminth transmission breakpoints are themselves complex dynamic entities influenced by the nature and magnitude of vector- and host-specific density-dependent processes, the local characteristics of vector competence and vectorial capacity in different vector species, the degree of parasite overdispersion among hosts in the population, and the interactions of these with the intervention(s) deployed [48], [63]–[65]. This highlights the problems faced in trying to obtain a single, “one size fits all”, infection breakpoint value that can be applied across a variety of epidemiological settings, suggesting that the end game in parasite elimination programmes will have to respond flexibly and adaptively to the locale-specific microepidemiology of infection in endemic communities [66].

### Models for Investigation of Evolutionary Outcomes of Control

Recently, mathematical models of parasite population dynamics such as those summarised in Table S1 have been modified to incorporate parasite genetic structure with regard to drug susceptibility [67]–[69]. These models have permitted, for example in the filarial nematodes, theoretical exploration of the spread of putative resistance alleles under various assumptions of the genetics of drug resistance (how many loci would be involved, whether or not these are linked [inherited together], whether anthelmintic resistance is conferred by recessive alleles) and parasite inbreeding.

As part of the M&E strategies, models can also assist, in critical ways, in the design of treatment efficacy and effectiveness studies; phenotypic characterisation of responses to treatment [70]; and design of sampling protocols for the study of parasite genetic structure under treatment, thereby facilitating prompt detection of anthelmintic resistance [71].

With regard to schistosomes, a model including time delays, mating structure, multiple resistant strains, and additional biological complexity associated with the parasite's life cycle has been used to explore the impact of drug treatment on resistant strain survival. This model suggests that time delays make it more likely for drug-resistant strains to spread in a parasite population [72], [73]. Other models, in which resistance has a fitness cost, in terms of reduced reproduction and transmission, have been used to infer the impact of drug treatment on the maintenance of schistosome genetic diversity. The likelihood that resistant strains will increase in frequency depends on the interplay between their relative fitness, the cost of resistance, and the degree of selection pressure exerted by the drug treatments [72], [74].

## Research Gaps in Helminth Modelling

Most of our understanding of parasite population biology and subsequent modelling efforts have focused on the study of endemic equilibrium situations. Frameworks that explore the impact of control scenarios do so based on the same assumptions made when describing the behaviour of the host–parasite systems at such endemic equilibrium, and for genetically homogeneous populations. In only a few of the more recent studies have models been fitted to data systematically collected during the interventions. Such studies have been possible because the programmes using these models have had the foresight and resources to undertake substantial longitudinal cohort studies [57]. Only very recently have studies incorporating genetic data on parasite variability begun to be undertaken [75], [76]. Although it is easy to understand why such robust and critical studies are only now being conducted (the impetus for global parasite control and elimination efforts has truly gained momentum in the 21st century, along with the explosion in genetics), we hold that the need for such well planned and resourced studies is critical to the success of parasite control efforts. As we enter the second decade of the 21st century, and despite global financial difficulties, we hope to move into a period of sustained parasite control and elimination where feasible. However, it is widely recognised that current programmes rely on few tools, predominantly a very limited arsenal of affordable or donated drugs (whose modes of action and modes of resistance are, for the most part, unknown), making such programmes particularly vulnerable to the development of anthelmintic resistance [77].

Critically needed is a renewed focus on the processes that determine reinfection; investigation of the long-term impact of changes in exposure and parasite acquisition/mortality on host immune response; an exploration of the prolonged effects of anthelmintics on the biology (and particularly the reproductive biology and mating structure) of the parasites in question; and finally, improved understanding of the relationship between infection and disease. Such understanding will enhance the efficacy and effectiveness of programmes that aim at morbidity control.

For those programmes that aim at elimination, a priority is the development and validation of models that account for the decreased sensitivity of currently available, and often inadequate, diagnostic tests (see companion review [78]). Such models will aid the interpretation of complementary serological (antibody and antigen) measures in study populations, quantify the contribution to transmission of ultra-low parasite densities, and of major importance, inform surveillance sampling protocols. Also, the synergistic effects of adjuvant chemical and non-chemical means of parasite control (including vector and snail control, mop-up strategies, environmental modification, and health education) is an approach that should be explored and exploited [77]. Mathematical models have a greater potential than has been realised to date to provide evidence-based decision-making tools to support anthelmintic control programmes. In order to fully realize this potential, a greater disposition for dialogue and mutual understanding is needed between the architects of such programmes, their implementers in endemic countries, and the mathematical and population biologists developing the models (Box 2).

**Box 2. Summary Points for Mathematical Modelling of Helminthiases**

- Mathematical modelling should be embedded in the global research agenda for human helminth infections, as it has the potential to guide all stages of helminth control and elimination efforts, from their design and implementation, monitoring and evaluation, to post-control surveillance
- At present, this potential has not been fully realised in the area of helminth epidemiology and control, in contrast with other parasitic and infectious diseases (e.g., malaria, HIV)
- A major limitation is the lack of coherent and harmonised frameworks for the collating, curating, and sharing of databases from longitudinal studies and large-scale helminth control programmes for their use by modellers
- In turn, helminth modellers should commit to a collective effort encompassing both common questions and different modelling approaches enabling key issues in helminth epidemiology and control to be investigated collaboratively, yet from different analytical perspectives, using the best available data
- A continuous dialogue between modellers/statisticians and users/stakeholders would iron out many difficulties and help realise the potential of models to become fully embedded into parasite control strategies

### Co-Infections, Multiple Populations, and Niche Shifts

Although many of the populations afflicted by helminthiases are polyparasitised or co-infected with other pathogen species, most models consider the dynamics of single-species parasite populations. The majority of models also ignore spatial structure and are confined to closed populations of hosts, parasites, and vectors. More recently, however, models for investigation of the population dynamic consequences of co-infections [79], [80], of multiple, spatially heterogeneous populations [81], and of connected (meta-)populations [82] are starting to receive attention. The further development of these frameworks will constitute important scientific advances for our understanding of the effects of interventions affecting some parasite/vector species or zoonotic reservoirs more strongly than others; the effectiveness of integrated neglected tropical disease (NTD) control; and the ability of some parasites, pathogens, intermediate hosts, or vectors to invade/occupy niches previously used by those species that are most vulnerable to particular interventions.

### Infection and Disease Mapping

Epidemiological and risk mapping integrates observed, georeferenced data and predictive, remote-sensing-derived environmental variables into model-based geostatistical approaches to indicate areas with different probabilities of infection presence and severity across chosen geographical scales, aiding national control programmes to evaluate the extent of the public health problem posed by helminth infection and deploy appropriate anthelmintic strategies [83]–[88]. Readers are referred to the Global Atlas of Helminth Infections (which at present provides an open-access information resource on the distribution of STHs and schistosomiasis in Africa) at http://www.thiswormyworld.org/.

The effectiveness of integrated NTD control programmes depends on the degree of geographical overlap between such diseases. However, in spite of being co-endemic at the country level, different helminth species may in certain settings exhibit limited geographical overlap at sub-national scales, necessitating a more geographically targeted approach [89], [90]. Thus, it will be important to devise optimal strategies for rapidly and simultaneously assessing the epidemiology of multiple helminth infections so as to effectively implement integrated control approaches. In addition to mapping single infections, risk and prediction of co-infection mapping should be developed to aid integrated and cost-effective control [91], [92]. Efforts should also be devoted to linking statistical epidemiological mapping with dynamic epidemiological modelling such that the outcomes of interventions over various geographical scales can be simulated and their impact evaluated.

### Morbidity Control and Elimination of Helminthiases as a Public Health Problem

A major gap in helminthiases research is the development of statistical and dynamic models linking infection and morbidity. Rigorous evaluation of programmes aiming at elimination of helminthiases as a public health problem hinges on assessing the point at which infection levels have been reduced below those that no longer represent a disease burden to the individual or the population. This is an area of ongoing and much needed research. Recent progress has been made on the use of statistical modelling to ascertain the relationship between microfilarial load and blindness incidence as well as excess mortality in onchocerciasis [93], [94], and the relationship between infection and morbidity indicators in schistosomiasis [95]. In dynamic models, morbidity has been modelled as a variable depending on the density and distribution of adult worms [29], [31] or of transmission stages (eggs or larvae) [2], depending on which stages are responsible for most pathology. More research is needed to ascertain how morbidity relates to present, lagged, and/or cumulative experience of infection and co-infection, and to link dynamical models of infection and disease into the estimation of disease burden and cost-effectiveness analysis of interventions.

Cost-effectiveness analysis using parameterised dynamic infection models enables the long-term effectiveness of an intervention to be estimated [96]–[98], avoiding the limitation of so-called static economic models [97], which consider only the effectiveness of an intervention at a particular point in time (e.g., [99], [100]) or over a limited period of follow-up (e.g., [101], [102]). This permits a more comprehensive assessment of effectiveness and allows interventions that elicit different dynamics to be compared fairly. For example, ivermectin (a microfilaricide) elicits a pronounced yet transient reduction in the numbers of *Onchocerca volvulus* microfilariae in human skin [52], while doxycycline (a macrofilaricide) causes a gradual but sustained reduction [103]. A fair comparison of the effectiveness of these drugs must account for the markedly different durations over which they act. Linking infection models to the prevalence of disease and associated morbidities [29], [30] further improves the capacity to capture the full benefits of an intervention [97], [98], and the predictive capability of models permits a priori comparison of a range of intervention strategies under various scenarios [96], [98]. Cost-effectiveness analysis using dynamic infection models needs to be further developed and made more accessible as a decision-making tool for the planners and implementers of control initiatives.

### Models for Assessing the Impact of Climate Change

Although it is well recognised that the transmission of helminthiases is strongly conditional on biotic and abiotic environmental factors, the latter including temperature, relative humidity, rainfall patterns, and hydrology, there are scarce data documenting the effects of these factors on life history traits of parasites, vectors, and intermediate hosts. Therefore, the impact of environment-driven changes on population dynamics and direct and indirect effects on transmission is poorly understood. This incomplete mechanistic understanding of environment–helminth disease interactions is reflected in the fact that mathematical models for such diseases have seldom included the effects of environmental processes on transmission dynamics. Recent modelling work on schistosomiasis japonica is addressing these deficiencies [104]–[106], which constitute an important research gap in the modelling of human helminthiases.

Linked to an increased awareness of the environmental determinants of infectious disease transmission, the effect of global warming on human health is an important topic that has received much interest in recent years. However, the precise effects of climate change on vector competence, duration of extrinsic incubation periods, survival of vectors, intermediate hosts, and reservoirs, and parasite transmission cycles in general remain poorly understood for the helminthiases [107].

There are two principal strategies for managing or reducing the risks of environmental change: mitigation and adaptation. The former seeks to reduce the presence and strength of anticipated risk factors (when these are known). The latter accepts that some degree of environmental change is inevitable and seeks to limit its negative impacts by encouraging and investing in preparedness. Both mitigation and adaptation will require detailed assessment of the existing distribution of the infections, their vectors, and intermediate/definitive hosts, and of the environmental determinants to which these are sensitive, including temperature, humidity, rainfall, vegetation cover, changes in the distribution and nature of water bodies, and modifications to agricultural and husbandry practices, among others [108]. The combined impact of these determinants on the transmission cycles of and rates of exposure to helminth infections of humans is poorly understood, and relevant information remains scattered in the literature, calling for systematic phenology reviews and experimental investigation. The results of these will help parameterise models with which to predict the consequences of climate change on the incidence and severity of human helminthiases. Among the few available modelling studies are those assessing the potential impact of rising temperature on the transmission of schistosomiasis [109]–[112]. Table S1 reveals a striking paucity of models for the transmission dynamics, control, and morbidity due to cestode infections, which needs to be addressed in light of climate change and its impact on agricultural and farming practices.

## A Research and Development Agenda for the Mathematical Modelling of Helminth Infections of Humans

In view of the historical (summarised above) and recent (Table S1) advances in mathematical modelling, and the identified research gaps and priorities for helminth epidemiology and population biology (Table S2), and mathematical modelling (Table S3), a research and development agenda requires the development of models that will be essential to advance helminthiasis control. Such models will lead to the identification of novel tools, critical research, and programmatic approaches that will be required for elimination of the public health problem posed by these infections or the infection reservoirs themselves (Box 3). Models will be essential for:

- estimating the impact of large-scale interventions on the incidence of infection and disease;
- designing sampling protocols for the M&E of integrated control programmes;
- facilitating the understanding of co-infections;
- investigating the relationship between infection and morbidity;
- improving analytical methods for the quantification of anthelmintic efficacy and resistance;
- determining programme end points;
- linking dynamic helminth models with helminth geostatistical mapping; and
- investigating the impact of environmental and climate change drivers on human helminthiases.

**Box 3. Research and Development Agenda for Modelling**

Few mathematical models have been effectively used to support decisions in the context of the implementation and evaluation of helminth control programmes. To realize the full potential of policy-relevant models, it will be necessary to:

- **Fit models to longitudinal data:**
  - Estimate changes in exposure and force of infection (incidence) to evaluate impact of control programmes and refine control strategies (frequency and duration of interventions)
  - Evaluate temporal trends and modalities of treatment frequency, duration, coverage, adherence, and their impact on transmission and infection
  - Analyse longitudinal immuno-epidemiological studies to investigate the impact of anthelmintic treatment on the strength and duration of immune responses
- **Aid the design of sampling protocols for monitoring and evaluation and surveillance** particularly for the integrated control of co-infecting neglected tropical diseases
- **Develop and validate mathematical models for co-infections** to ascertain how control/elimination goals may be altered by synergistic/antagonistic interactions between helminths (or between helminths and other parasites) in polyparasitised populations
- **Refine models for the relationships between infection and morbidity indicators** that take into account present and cumulative effects for evaluation of disease burden and the impact on such burden of control interventions
- **Develop further cost-effectiveness analysis using dynamic infection models** as a decision-making tool for planners and implementers of control initiatives
- **Guide assessment of anthelmintics efficacy and effectiveness:**
  - Improve current quantitative methods to measure drug efficacy
  - Identify factors involved in the manifestation of well-characterised suboptimal responses to treatment, including drug resistance and non-parasite genetic factors
  - Develop models linking parasite phenotypic and genotypic data regarding treatment responses
  - Develop models merging helminth population biology and population genetics to investigate the spread and mitigation of anthelmintic resistance
- **Investigate and determine end points and transmission breakpoints from programmatic viewpoints:**
  - Integrate models with data to explore the dynamics of transmission breakpoints for the host–parasite combinations prevailing in endemic areas
  - Use modelling to update and refine assumed elimination thresholds
- **Link Bayesian geostatistical mapping with dynamic helminth models** to simulate interventions alone or in combination and evaluate their impact at different geographical scales and endemicity levels
- **Develop models for investigation of climate change on helminth infections and their control:**
  - Conduct literature reviews, and experimental/observational studies and parameter estimation
  - Develop and calibrate models taking into account the interaction between the biology of the infection and climate-driven environmental variables

## Compilation, Curation, and Sharing of Databases

An essential prerequisite for the advancement of control through the use of modelling tools, is that of high quality, openly accessible data. In this respect, a major impediment to the development of modelling as a useful decision-support tool for helminth control programmes is the lack of coherent and harmonised frameworks for the collating, annotating, curating, and sharing of databases from helminth control programmes and reinfection studies, past and present, for their use by the community of modellers. Some steps in the right direction can be seen in initiatives such as the Global NGO Deworming Inventory, which collects treatment data from nongovernmental organisations (NGOs) around the world that provide anthelmintic drugs to treat STHs, schistosomiasis, and/or LF (http://www.deworminginventory.org/), and the World Health Organization (WHO) Preventive Chemotherapy (PCT) data bank, which collects treatment data from governmental health agencies (http://www.who.int/neglected_diseases/preventive_chemotherapy/databank/en/index.html). Databases such as those of the OCP, the African Programme for Onchocerciasis Control (APOC), the Onchocerciasis Elimination Program for the Americas (OEPA) (not included in the above-mentioned WHO data bank), the Schistosomiasis Control Initiative (SCI), and the Global Programme to Eliminate Lymphatic Filariasis (GPELF), among others, should be made openly accessible and available by the custodians of those data, under mutually agreeable protocols, to a broad diversity of modelling groups to facilitate the application of a variety of quantitative approaches for the resolution of key epidemiological and operational questions. This, in turn, will facilitate the dissemination of model outputs to the community of users, stakeholders, and contributors to the collection of such data. For this to be achieved, helminth modellers should commit to a collective effort, encompassing both common questions and complementary modelling approaches to enable key issues in helminth population biology and control to be investigated collaboratively, yet from different analytical perspectives, and using the best available data.

Examples of such initiatives exist in the areas of mathematical modelling of other infections, such as that proposed by the malERA Consultative Group on Modelling for malaria eradication [113], and MIDAS (Models of Infectious Disease Agent Study) for the modelling of emerging infectious diseases and outbreaks [114], and are partly responsible for the increasing success of mathematical modelling in influencing public health policy and practice to a much greater degree than it has been possible to date in tackling the problem of helminthiasis [115].

## Conclusions and the Challenge of Engaging Multiple Actors

A closer collaboration between biometrician and [parasitologist], and a better acquaintanceship of each with the methods of the other, is one of the most useful things we can work for today. —L. W. Hackett (1937) [116].

Helminth infections affect disproportionally, and impose their highest burdens on the least privileged and most impoverished populations of the planet. Yet, very few mathematical models have been used by policy-makers to support evidence-based decisions in the context of the implementation and M&E of helminth control programmes. The reasons for this missed opportunity are multifarious, but they must be understood and overcome if the full potential of policy-relevant models and modelling studies in general is to be realised. On the one hand, model outputs may not be easily interpretable to the non-expert, or may not have a direct relationship with the assays/indicators (and their limitations) that are used by the control programmes to monitor intervention progress. Policy-makers may be influenced by the political need to demonstrate success, in the face of modelling outputs highlighting concerns. The clear-cut answers that may be demanded or required may not be met by models, and, importantly, the implications of model assumptions and uncertainties may not be fully appreciated.

On the other hand, modellers may not have made sufficient efforts to communicate their findings to non-specialised audiences, and to translate model outputs into readily understood and epidemiologically relevant measures of infection and morbidity. There is a clear need to create user-friendly interfaces for advocacy, education, and ease of application by end users. There is a risk that field workers may feel dispossessed when their hard-earned data are taken by modellers and repackaged into elegant publications that bear little relationship with reality, or which fail to appropriately acknowledge the difficulties experienced by those collecting and collating the data. More importantly, in the context of parasite control, the questions explored by modellers may not be motivated or sharpened by the needs of the stakeholder and end user (scientific and other) communities. This brings the challenge of engaging multiple actors to the fore. There is a risk that empiricists may not appreciate the potential contribution of modellers, considering them as theoreticians; modellers may risk simplification of biological complexity to facilitate model tractability; and the programmes seeking “magic bullets” may be frustrated by the inherent uncertainty of model outputs.

Notwithstanding these difficulties, a continuous dialogue between quantitative epidemiologists and those implementing control programmes is essential. If modellers and statisticians are involved from the outset during the early phases of funding applications, programme design and implementation, and subsequent M&E, unrealised value will accrue, including the resolution of the many difficulties that inevitably will arise. This approach will help realise the potential of models that are fully embedded into control and elimination strategies to greatly facilitate the control of the helminth infections of humankind.

## Supporting Information

Figure S1 Basic and Effective Reproduction Ratios
(PDF)

Table S1 Summary of Mathematical Models for Human Helminthiases
(PDF)

Table S2 Gap Analysis for Helminth Epidemiology
(PDF)

Table S3 Gap Analysis for Mathematical Models
(PDF)

Text S1 Facilitation and Limitation
(PDF)

Text S2 Basic and Effective Reproduction Ratios
(PDF)
