# Supplementary material for: A Research Agenda for Helminth Diseases of Humans: Modelling for Control and Elimination
Source: PLoS Negl Trop Dis. 2012 Apr 24;6(4):e1548. doi: 10.1371/journal.pntd.0001548 (PMC3335861; doi:10.1371/journal.pntd.0001548)
Supplement: Figure S1 — Basic and Effective Reproduction Ratios (PDF) [file pntd.0001548.s001.pdf]

Figure S1. The Basic and Effective Reproduction Ratios of Helminth Infections (for definitions see Text S2)

A. The effective reproduction ratio (R_E) plotted against skin microfilarial load (mf/mg) for the onchocerciasis model of [1], assuming that all adult *Onchocerca volvulus* female worms are mated and incorporating (negative) density dependence of parasite establishment within humans and vectors and parasite-induced vector mortality, for three villages in Cameroon with increasing annual biting rate by *Simulium damnosum*: Tchollir é (ABR=1,000 bites per person per year, blue); Douffing (ABR=2,507, green); Touboro (ABR=8,960, red). Because facilitating density dependence has not been included, the only (non-trivial) equilibrium of the system is locally and globally stable and corresponds to the endemic parasite density, at which R_E crosses 1 (Tchollir é, predicted = 11, observed = 10 mf/mg; Douffing, predicted = 36, observed = 21 mf/mg; Touboro, predicted = 58, observed = 56 mf/mg). In the absence of an unstable equilibrium, the maximum value of R_E corresponds to the basic reproduction ratio (R_0), reached as microfilarial load tends to zero. For the three villages the estimated values of R_0 are1.5, 3.8 and 13.2 respectively.

B. The effective reproduction ratio (R_E) plotted against mean adult worm burden for the model of [2], parameterised for *Ascaris lumbricoides*, taking into account the mating probability (a positive density-dependent process) and density-dependent worm fecundity (a negative density-dependent process) at three levels of severity; blue, 0.01 per worm indicating light constraints on worm fecundity; green, 0.1 per worm, illustrating moderate constraints; red, 0.3, corresponding to stronger constraints. The inclusion of facilitation (mating probability) gives rise to unstable equilibria (transmission breakpoints) to the left-hand side of the maximum (hump) R_E value (where R_E crosses 1, represented by dotted lines, respectively, 0.44, 0.06, and 0.03 worms per person for increasing severity of negative

density dependence). The point at which R_E crosses 1 on the right-hand side is the stable, endemic equilibrium (represented by the black dotted line at 14.5 worms per person). The values of peak R_E represent the maximum transmission potential of the parasite (since R_0 is density-independent it does not pertain here), and the values are 1.23, 2.61, and 4.14 (blue, green, and red dashed lines).

References

1. Basáñez MG, Boussinesq M (1999) Population biology of human onchocerciasis. Phil Trans R Soc Lond B 354: 809–826.
2. Churcher TS, Filipe JAN, Basáñez MG (2006) Density dependence and the control of helminth parasites. J Anim Ecol 75: 1313–1320.

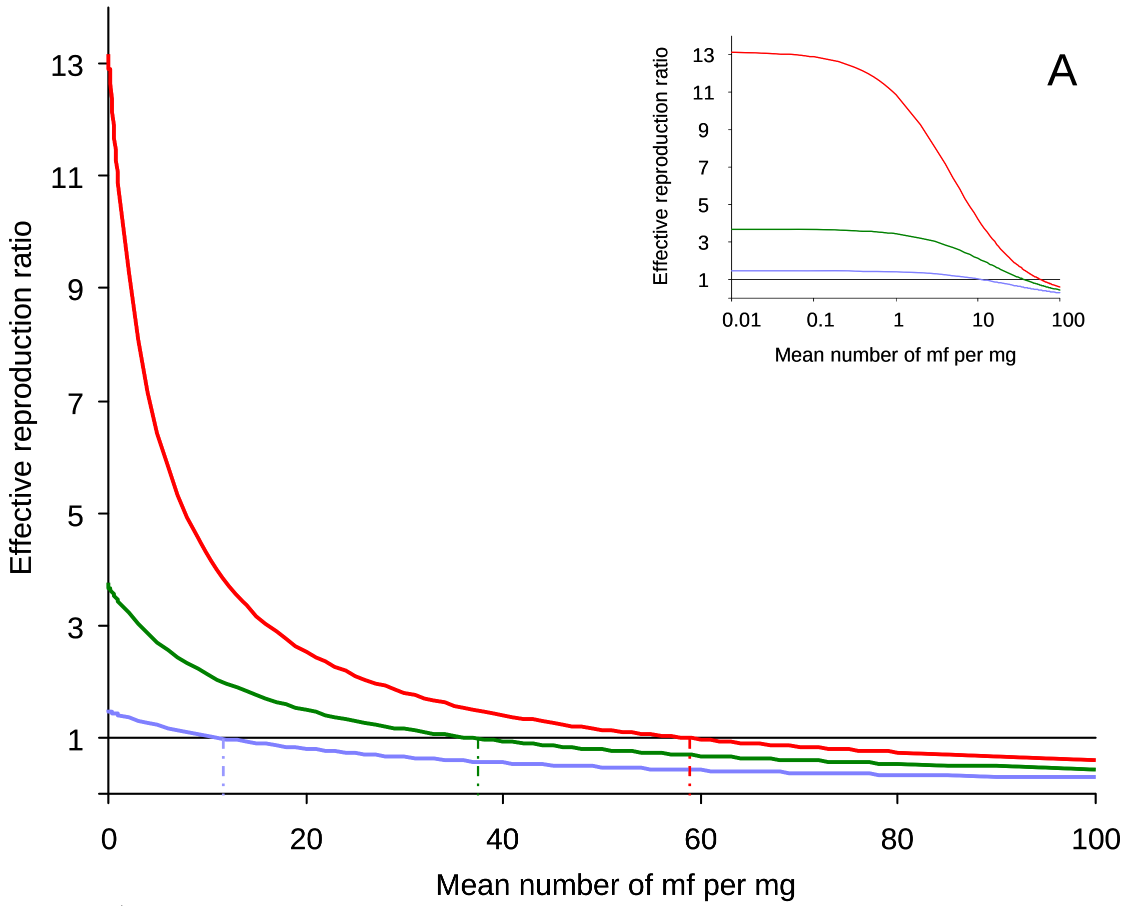
A
Effective reproduction ratio
13
11
9
7
5
3
1
0
20
40
60
80
100
Mean number of mf per mg
0.01
0.1
1
10
100

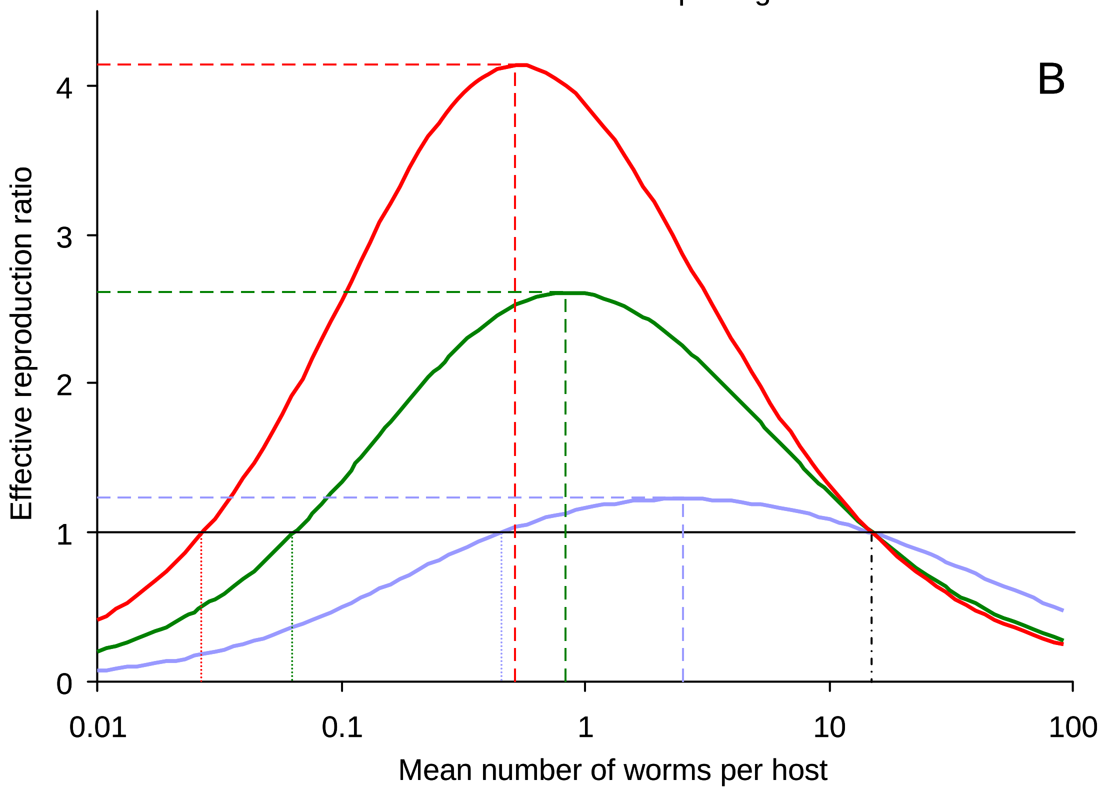
B
Effective reproduction ratio
4
3
2
1
0
0.01
0.1
1
10
100
Mean number of worms per host
