# Supplementary material for: A Research Agenda for Helminth Diseases of Humans: Modelling for Control and Elimination
Source: PLoS Negl Trop Dis. 2012 Apr 24;6(4):e1548. doi: 10.1371/journal.pntd.0001548 (PMC3335861; doi:10.1371/journal.pntd.0001548)
Supplement: Table S1 — Summary of Mathematical Models for Human Helminthiases (PDF) [file pntd.0001548.s002.pdf]

Table S1. A Summary of Mathematical Models for Helminth Infections of Humans

Helminth infection and causal agent	Role of model	Type of model and framework	Questions explored	References
Soil-transmitted helminthiases, in particular ascariasis and hookworm infection	Population biology & transmission dynamics	Deterministic; infection intensity	Role of density-dependent fecundity	[1,2]
		Stochastic	Mode of transmission of infective stages, trickle or clumped	[3]
	Exploration of intervention scenarios	Deterministic, age-structured, intensity and morbidity	Compare impact of MDA and age-targeted chemotherapy on morbidity	[4,5] (EpiWorm)
		Stochastic, individual-based model	Assess the relative impact of chemotherapy and vaccination against human hookworm infection	[6]
Intestinal and urinary schistosomiasis due to *Schistosoma mansoni* and *S. haematobium*	Population biology & transmission dynamics	Deterministic; prevalence of infection in humans and snails	Use of models for evaluating possible control strategies; limitations and uncertainties	[7]
		Deterministic; infection intensity in humans; infection prevalence in snails	Heterogeneities; snail dynamics; miracidia & cercaria dynamics; acquired immunity	[8,9]
		Stochastic	Investigation of the role of immunity in observed patterns in endemic communities	[10,11]
	Exploration of intervention scenarios	Deterministic; infection intensity in humans	Chemotherapy; vaccination; snail control; larval stage control; improved water & sanitation; health education	[12–15]
			Dynamical aspects of the interactions between treatment, immunity and morbidity and consequences for control programmes	[16–18]
	Population biology & exploration of control scenarios	Deterministic; age-structured; infection intensity and prevalence in humans; prevalence of heavy infections	Phenomenological representation of acquired immunity; MDA vs. age-targeted chemotherapy; impact on morbidity	[16,19–21] (EpiSchisto)
		Deterministic; infection intensity in humans and prevalence in snails	Infected snails suffer excess mortality and no reproduction	[22]
	Fitting models to data; estimation of transmission parameters	Deterministic; infection intensity in humans	Changes in the force of infection after annual treatment in Uganda	[23]
	Inclusion of human-worm- and infection-related aspects	Stochastic; lacking transmission-related mechanisms	Short-term effects of schistosome surveys & treatments	[24] (SCHISTOSIM)
	Exploring the evolutionary outcomes of control; drug resistance in schistosomiasis	Differential equations, time delays, mating structure	Exploration of the role of mating structure in the maintenance of genetic diversity	[25–27]

Table S1. Continued

Helminth infection and causal agent	Role of model	Type of model and framework	Questions explored	References
Zoonotic schistosomiasis, *S. japonicum*	Population biology & exploration of control scenarios	Deterministic; prevalence of infection in humans and bovines	MDA vs. targeted treatment of high prevalence groups of humans and bovines	[28,29]
		Infection intensity in humans; infection prevalence in snails; connected villages	Relative roles of environmental and social connectivity for parasite spread, persistence and control	[30]
	Population biology & exploration of control scenarios. Estimation of transmission parameters	Infection intensity in humans (subdivided by occupational and residence groups); prevalence in snails	Occupation-targeted chemotherapy; snail control; environmental aspects of contamination and exposure	[31–34]
		Prevalence of infection in humans, rodents and snails	Chemotherapy; snail control / molluscicides; elimination from Bohol Island, Philippines	[35]
	Fitting model to data. Estimation of transmission parameters	Humans stratified by sex, age, and intensity class<br>Infection prevalence for a range of hosts and villages	Relative importance of transmission from different mammals to snails and from snails to mammals	[36,37]
	Transmission dynamics and its interaction with environmental variables	Coupling a hydrological model with a delay-differential equation schistosomiasis transmission model	Influence of hydrological factors on the prevalence, intensity and timing of infection; influence of hydrologic variability greater on the cercarial stage of the parasite than the miracidial stage	[38]
Onchocerciasis	Population biology & transmission dynamics at endemic equilibrium	Deterministic; infection intensity in humans and simuliid vectors	Role of density-dependent parasite establishment in humans and vectors; propensity of vectors to feed on humans; density-dependent host choice; threshold biting rates in Africa and Latin America	[39–42]
	Exploration of intervention scenarios	Deterministic; infection intensity in humans and simuliid vectors	Impact of nodulectomy alone vs. nodulectomy plus mass ivermectin administration	[43] (also a review of onchocerciasis models)
	Fitting model to data; estimation of (exposure) parameters	Deterministic; host age- and sex-structured	Differential age- and sex-specific exposure to vectors according to human ecology	[44]
	Investigation of ivermectin resistance	All of the above plus parasite genetic-structure regarding ivermectin susceptibility	Sampling protocols to maximize detection of ivermectin resistance	[45]

Table S1. Continued

Helminth infection and causal agent	Role of model	Type of model and framework	Questions explored	References
Onchocerciasis	Transmission dynamics; exploration of intervention scenarios; feasibility of parasite elimination	Stochastic for parasites and humans; deterministic for vectors	Duration of vector control; combination of vector control and mass ivermectin distribution for control and elimination	[46–49] (ONCHOSIM)<br><br>[50] (SIMON, West Africa; SIMON-A, Americas)
	Population biology at endemic equilibrium	Stochastic	Adult worm aggregation in nodules; density-dependent parasite establishment and fecundity; threshold biting rates in Africa	[51–54]
	Exploration of intervention scenarios	Deterministic; exposure heterogeneity in the human population	Exploration of mass vs. targeted ivermectin distribution	[55]
	Investigation of transmission breakpoints	Full transmission model with positive and negative density-dependent processes	Factors determining the magnitude of transmission breakpoints (vector density, vector competence, propensity to feed on humans) and interactions with treatment	[56,57]
	Investigation of ivermectin effects and efficacy on adult worms and microfilariae	Deterministic; adult female worm population structured in fecund and non-fecund worms	Dynamics of microfilarial reappearance in skin and anti-fecundity effects after single dose treatment	[58]
	Investigation of sub-optimal responses to ivermectin	Individual-based model	Variability in rates of microfilarial reappearance in the skin between populations ivermectin naïve and multiply treated	[59]
	Investigation of anthelmintic resistance	Deterministic; over-dispersed parasite distribution; genetic structure	Resistance allele frequencies in different life-stages following treatment	[60]<br><br>(also for STHs)
Lymphatic filariasis	Population biology & transmission dynamics; infection and morbidity control; endpoints; monitoring & evaluation	Deterministic; host age-structured, infection intensity in humans and vectors	Morbidity reduction; treatment with drugs exerting different effects on adult worms and microfilariae; vector control; programme endpoints	[61–65]<br><br>(EpiFil)
	Investigation of transmission breakpoints	Deterministic and stochastic frameworks	Factors determining local variation in elimination thresholds, dependent on vector composition, vector density and initial endemicity	[66,67]
	Population biology & transmission dynamics; exploration of control scenarios	Stochastic	Role of acquired immunity; development of morbidity; effects of control measures (drug treatment and/or mosquito control)	[68–70]<br><br>(LYMFASIM)
	Investigation of benzimidazole and ivermectin resistance	Deterministic; host age- and parasite genetically-structured, based on EPIFIL	Role of parasite inbreeding; linked resistance; interpretation of molecular genetics surveys	[71–73]

Table S1. Continued

Helminth infection and causal agent	Role of model	Type of model and framework	Questions explored	References
Taeniasis, cysticercosis, and hydatic disease	Exploration of intervention scenarios for *Taenia solium*	Deterministic and stochastic versions of a modified Reed-Frost model	(i) Use of latrines, meat inspection, cooking habits; (ii) rapid detection and treatment of human carriers; pig vaccination; (iii) treatment of humans or pigs	[74]
	Evaluation of control programmes against *Taenia solium* cysticercosis	Stochastic simulation model	Effect of treatment of humans and pigs (alone or in combination) linked to economic evaluation	[75]
	Population dynamics of *Echinococcus granulosus* and exploration of control interventions	Mathematical models of the transmission dynamics between dogs and livestock for Australia, New Zealand, Middle East and Asia	(i) Intense anthelmintic treatment of the dog population; (ii) introduction of newly developed sheep vaccine	[76]
	Population dynamics in echinococcosis and cysticercosis	Integro-differential equations	Population dynamics of *Taenia hydatigena* and *T. ovis*; role of cross immunity and impact of control	[77,78]

The need to develop mathematical frameworks for food-borne trematodiasis transmission under a range of conditions, enabling identification of opportunities for implementation of control measures, has been expressed [79]. However, no mathematical models have yet been developed for the transmission and control of *Clonorchis sinensis*; *Opisthorchis viverrini*, *Opisthorchis felineus* or *Paragonimus* spp. Consideration of models for fasciolasis is outside the remit of the Disease Reference Group for Helminth Infections.

References

1. Anderson RM (1982) The population dynamics and control of hookworm and roundworm infections. In: *Population Dynamics of Infectious Diseases* (ed. Anderson RM), pp. 67–108. London: Chapman and Hall.

2. Anderson RM, Medley GF (1985) Community control of helminth infections of man by mass and selective chemotherapy. Parasitology 90: 629–660.

3. Walker M, Hall A, Basáñez MG (2010) Trickle- or clumped infection process? A stochastic model for the infection process of the parasitic roundworm of humans, *Ascaris lumbricoides*. Int J Parasitol 40: 1381–1388.

4. Medley GF, Guyatt HL, Bundy DAP (1993) A quantitative framework for evaluating the effect of community treatment on the morbidity due to ascariasis. Parasitology 106: 211–221.

5. Chan MS, Guyatt HL, Bundy DAP, Medley GF (1994) The development and validation of an age-structured model for the evaluation of disease control strategies for intestinal helminths. Parasitology 109: 389–396.

6. Sabatelli L, Ghani AC, Rodrigues LC, Hotez PJ, Brooker S (2008) Modelling heterogeneity and the impact of chemotherapy and vaccination against human hookworm. J R Soc Interface 5: 1329–1341.

7. Barbour AD (1996) Modeling the transmission of schistosomiasis: an introductory view. Am J Trop Med Hyg 55 (5 Suppl): 135–143.

8. Anderson RM, May RM (1985) Helminth infections of humans: mathematical models, population dynamics, and control. Adv Parasitol 24: 1–101.

9. Woolhouse MEJ (1991) On the application of mathematical models of schistosome transmission dynamics. I. Natural transmission. Acta Trop 49: 241–270.

10. Chan MS, Isham VS (1998) A stochastic model of schistosomiasis immuno-epidemiology. Math Biosci 151: 179–198.

11. Chan MS, Mutapi F, Woolhouse MEJ, Isham VS (2000) Stochastic simulation and the detection of immunity to schistosome infections. Parasitology 120: 161–169.

12. Woolhouse MEJ (1992) On the application of mathematical models of schistosome transmission dynamics. II. Control. Acta Trop 50: 189–204.

13. Woolhouse MEJ (1995) Human schistosomiasis: potential consequences of vaccination. Vaccine 13: 1045–1050.

14. Woolhouse MEJ (1996) Mathematical models of transmission dynamics and control of schistosomiasis. Am J Trop Med Hyg 55 (5 Suppl): 144–148.

15. Chan MS, Woolhouse MEJ, Bundy DAP (1997) Human schistosomiasis: potential long-term consequences of vaccination programmes. Vaccine 15: 1545–1550.

16. Chan MS, Anderson RM, Medley GF, Bundy DAP (1996) Dynamic aspects of morbidity and acquired immunity in schistosomiasis control. Acta Trop 62: 105–117.

17. Chan MS, Guyatt HL, Bundy DAP, Medley GF (1996) Dynamic models of schistosomiasis morbidity. Am J Trop Med Hyg 55: 52–62.

18. Chan MS, Bundy DAP (1997) Modelling the dynamic effects of community chemotherapy on patterns of morbidity due to *Schistosoma mansoni*. Trans R Soc Trop Med Hyg 91: 216–220.

19. Medley GF, Bundy DAP (1996) Dynamic modeling of epidemiologic patterns of schistosomiasis morbidity. Am J Trop Med Hyg 55 (5 Suppl): 149–158.

20. Chan MS, Nsowah-Nuamah NN, Adjei S, Wen ST, Hall A, Bundy DAP (1998) Predicting the impact of school-based treatment for urinary schistosomiasis given by the Ghana Partnership for Child Development. Trans R Soc Trop Med Hyg 92: 386–389.

21. Chan MS, Guyatt HL, Bundy DAP, Booth M, Fulford AJ, Medley GF (1995) The development of an age structured model for schistosomiasis transmission dynamics and control and its validation for *Schistosoma mansoni*. Epidemiol Infect 115: 325–344.

22. Zhao R, Milner FA (2008) A mathematical model of *Schistosoma mansoni* in *Biomphalaria glabrata* with control strategies. Bull Math Biol 70: 1886–1905.

23. French MD, Churcher TS, Gambhir M, Fenwick A, Webster JP, Kabatereine NB, Basáñez MG (2010) Observed reductions in *Schistosoma mansoni* transmission from large-scale administration of praziquantel in Uganda: a mathematical modelling study. PLoS Negl Trop Dis 4: e897.

24. de Vlas SJ, Van Oortmarssen GJ, Gryseels B, Polderman AM, Plaisier AP, Habbema JD (1996) SCHISTOSIM: a microsimulation model for the epidemiology and control of schistosomiasis. Am J Trop Med Hyg 55 (5 Suppl): 170–175.

25. Xu D, Curtis J, Feng Z, Minchella DJ (2005) On the role of schistosome mating structure in the maintenance of drug resistant strains. Bull Math Biol 67: 1207–1226.

26. Castillo-Chavez C, Feng Z, Xu D (2008) A schistosomiasis model with mating structure and time delay. Math Biosci 211: 333–341.

27. Feng Z, Curtis J, Minchella DJ (2001) The influence of drug treatment on the maintenance of schistosome genetic diversity. J Math Biol 43: 52–68.

28. Williams GM, Sleigh AC, Li Y, Feng Z, Davis GM, Chen H, Ross AG, Bergquist R, McManus DP (2002) Mathematical modelling of schistosomiasis japonica: comparison of control strategies in the People's Republic of China. Acta Trop 82: 253–262.

29. Gray DJ, Williams GM, Li Y, McManus DP (2008) Transmission dynamics of *Schistosoma japonicum* in the lakes and marshlands of China. PLoS One 3: e4058.

30. Gurarie D, Seto EY (2009) Connectivity sustains disease transmission in environments with low potential for endemicity: modelling schistosomiasis with hydrologic and social connectivities. J R Soc Interface 6: 495–508.

31. Spear RC, Hubbard A, Liang S, Seto E (2002) Disease transmission models for public health decision making: toward an approach for designing intervention strategies for schistosomiasis japonica. Environ Health Perspect 110: 907–915.

32. Liang S, Maszle D, Spear RC (2002) A quantitative framework for a multi-group model of schistosomiasis japonicum transmission dynamics and control in Sichuan, China. Acta Trop 82: 263–277.

33. Liang S, Spear RC, Seto E, Hubbard A, Qiu D (2005) A multi-group model of *Schistosoma japonicum* transmission dynamics and control: model calibration and control prediction. Trop Med Int Health 10: 263–278.

34. Liang S, Seto EY, Remais JV, Zhong B, Yang C, Hubbard A, Davis GM, Gu X, Qiu D, Spear RC (2007) Environmental effects on parasitic disease transmission exemplified by schistosomiasis in western China. Proc Natl Acad Sci U S A 104: 7110–7115.

35. Ishikawa H, Ohmae H, Pangilinan R, Redulla A, Matsuda H (2006) Modeling the dynamics and control of *Schistosoma japonicum* transmission on Bohol island, the Philippines. Parasitol Int 55: 23–29.

36. Riley S, Carabin H, Marshall C, Olveda R, Willingham AL, McGarvey ST (2005) Estimating and modeling the dynamics of the intensity of infection with *Schistosoma japonicum* in villagers of Leyte, Philippines. Part II: intensity-specific transmission of *S. japonicum*. The schistosomiasis transmission and ecology project. Am J Trop Med Hyg 72: 754–761.

37. Riley S, Carabin H, Bélisle P, Joseph L, Tallo V, Balolong E, Willingham AL, Fernandez TJ, Gonzales RO, Olveda R, McGarvey ST (2008) Multi-host transmission dynamics of *Schistosoma japonicum* in Samar province, the Philippines. *PLoS Med* 5: e18.

38. Remais J, Liang S, Spear RC (2008) Coupling hydrologic and infectious disease models to explain regional differences in schistosomiasis transmission in southwestern China. Environ Sci Technol 42: 2643–2649.

39. Dietz K (1982) The population dynamics of onchocerciasis. In: *Population Dynamics of Infectious Diseases* (ed. Anderson RM), pp. 209–241. London: Chapman and Hall.

40. Basáñez MG, Boussinesq M (1999) Population biology of human onchocerciasis. Philos Trans R Soc Lond B 354: 809–826.

41. Basáñez MG, Collins RC, Porter CH, Little MP, Brandling-Bennett D (2002) Transmission intensity and the patterns of *Onchocerca volvulus* infection in human communities. Am J Trop Med Hyg 67: 669–679.

42. Basáñez MG, Razali K, Renz A, Kelly D (2007) Density-dependent host choice by disease vectors: epidemiological implications of the ideal free distribution. Trans R Soc Trop Med Hyg 101: 256–269.

43. Basáñez MG, Ricárdez-Esquinca J (2001). Models for the population biology and control of human onchocerciasis. Trends Parasitol 17: 430–438.

44. Filipe JAN, Boussinesq M, Renz A, Collins RC, Vivas-Martinez S, Grillet ME, Little MP, Basáñez MG (2005) Human infection patterns and heterogeneous exposure in river blindness. Proc Natl Acad Sci U S A 102: 15265–15270.

45. Churcher TS, Basáñez MG (2009) Sampling strategies to detect anthelmintic resistance: the perspective of human onchocerciasis. Trends Parasitol 25: 11–17.

46. Plaisier AP, van Oortmarssen GJ, Habbema JDF, Remme J, Alley ES (1990) ONCHOSIM: a model and computer simulation program for the transmission and control of onchocerciasis. Comput Methods Programs Biomed 31: 43–56.

47. Plaisier AP, van Oortmarssen GJ, Remme J, Alley ES, Habbema JDF (1991) The risk and dynamics of onchocerciasis recrudescence after cessation of vector control. Bull World Health Organ 69: 169–178.

48. Plaisier AP, Alley ES, van Oortmarssen GJ, Boatin BA, Habbema JDF (1997) Required duration of combined annual ivermectin treatment and vector control in the Onchocerciasis Control Programme in west Africa. Bull World Health Organ 75: 237–245.

49. Winnen M, Plaisier AP, Alley ES, Nagelkerke NJ, van Oortmarssen G, Boatin BA, Habbema JDF (2002) Can ivermectin mass treatments eliminate onchocerciasis in Africa? Bull World Health Organ 80: 384–391.

50. Davies JB (1993) Description of a computer model of forest onchocerciasis transmission and its application to field scenarios of vector control and chemotherapy. Ann Trop Med Parasitol 87: 41–63.

51. Duerr HP, Dietz K, Büttner DW, Schulz-Key H (2001) A stochastic model for the aggregation of *Onchocerca volvulus* in nodules. Parasitology 123: 193–201.

52. Duerr HP, Dietz K, Schulz-Key H, Büttner DW, Eichner M (2003) Density-dependent parasite establishment suggests infection-associated immunosuppression as an important mechanism for parasite density regulation in onchocerciasis. Trans R Soc Trop Med Hyg 97: 242–250.

53. Duerr HP, Dietz K, Schulz-Key H, Büttner DW, Eichner M (2004) The relationships between the burden of adult parasites, host age and the microfilarial density in human onchocerciasis. Int J Parasitol 34: 463–473.

54. Duerr HP, Eichner M (2010) Epidemiology and control of onchocerciasis: the threshold biting rate of savannah onchocerciasis in Africa. Int J Parasitol 40: 641–650.

55. Poolman EM, Galvani AP (2006) Modeling targeted ivermectin treatment for controlling river blindness. Am J Trop Med Hyg 75: 921–927.

56. Basáñez MG, Churcher TS, Grillet ME (2009) *Onchocerca-Simulium* interactions and the population and evolutionary biology of *Onchocerca volvulus*. Adv Parasitol 68: 263–313.

57. Duerr HP, Raddatz G, Eichner M (2011) Control of onchocerciasis in Africa: threshold shifts, breakpoints and rules for elimination. Int J Parasitol 41: 581–589.

58. Basáñez MG, Pion SDS, Boakes E, Filipe JAN, Churcher TS, Boussinesq M (2008) Effect of single-dose ivermectin on *Onchocerca volvulus*: a systematic review and meta-analysis. Lancet Infect Dis 8: 310–322.

59. Churcher TS, Pion SD, Osei-Atweneboana MY, Prichard RK, Awadzi K, Boussinesq M, Collins RC, Whitworth JA, Basáñez MG (2009) Identifying sub-optimal responses to ivermectin in the treatment of River Blindness. Proc Natl Acad Sci USA 106: 16716–16721.

60. Churcher TS, Basáñez MG (2008) Density dependence and the spread of anthelmintic resistance. Evolution 62: 528–537.

61. Chan MS, Srividya A, Norman RA, Pani SP, Ramaiah KD, Vanamail P, Michael E, Das PK, Bundy DAP (1998) EPIFIL: a dynamic model of infection and disease in lymphatic filariasis. Am J Trop Med Hyg 59: 606–614.

62. Norman RA, Chan MS, Srividya A, Pani SP, Ramaiah KD, Vanamail P, Michael E, Das PK, Bundy DAP (2000) EPIFIL: the development of an age-structured model for describing the transmission dynamics and control of lymphatic filariasis. Epidemiol Infect 124: 529–541.

63. Michael E, Malecela-Lazaro MN, Kabali C, Snow LC, Kazura JW (2006) Mathematical models and lymphatic filariasis control: endpoints and optimal interventions. Trends Parasitol 22: 226–233.

64. Michael E, Malecela-Lazaro MN, Kazura JW (2007) Epidemiological modelling for monitoring and evaluation of lymphatic filariasis control. Adv Parasitol 65: 191–237.

65. Michael E, Malecela-Lazaro MN, Maegga BT, Fischer P, Kazura JW (2006) Mathematical models and lymphatic filariasis control: monitoring and evaluating interventions. Trends Parasitol 22: 529–535.

66. Gambhir M, Michael E (2008) Complex ecological dynamics and eradicability of the vector borne macroparasitic disease, lymphatic filariasis. PLoS One 3: e2874.

67. Gambhir M, Bockarie M, Tisch D, Kazura J, Remais J, Spear R, Michael E (2010) Geographic and ecologic heterogeneity in elimination thresholds for the major vector-borne helminthic disease, lymphatic filariasis. BMC Biol 8: 22.

68. Plaisier AP, Subramanian S, Das PK, Souza W, Lapa T, Furtado AF, Van Der Ploeg CPB, Habbema JDF, Van Oortmarssen G J (1998) The LYMFASIM simulation program for modeling lymphatic filariasis and its control. Methods Inf Med 37: 97–108.

69. Subramanian S, Stolk WA, Ramaiah KD, Plaisier AP, Krishnamoorthy K, Van Oortmarssen GJ, Amalraj DD, Habbema JDF, Das PK (2004) The dynamics of *Wuchereria bancrofti* infection: a model-based analysis of longitudinal data from Pondicherry, India. Parasitology 128: 467–482.

70. Stolk WA, de Vlas SJ, Borsboom GJ, Habbema JDF (2008) LYMFASIM, a simulation model for predicting the impact of lymphatic filariasis control: quantification for African villages. Parasitology 135: 1583–1598.

71. Schwab AE, Churcher TS, Schwab AJ, Basáñez MG, Prichard RK (2006) Population genetics of concurrent selection with albendazole and ivermectin or diethylcarbamazine on the possible spread of albendazole resistance in *Wuchereria bancrofti*. Parasitology 133: 589–601.

72. Schwab AE, Churcher TS, Schwab AJ, Basáñez MG, Prichard RK (2007) An analysis of the population genetics of potential multi-drug resistance in *Wuchereria bancrofti* due to combination chemotherapy. Parasitology 134: 1025–1040.

73. Churcher TS, Schwab AE, Prichard RK, Basáñez MG (2008) An analysis of genetic diversity and inbreeding in *Wuchereria bancrofti*: implications for the spread and detection of drug resistance. PLoS Negl Trop Dis 2: e211.

74. Kyvsgaard NC, Johansen MV, Carabin H (2007) Simulating transmission and control of *Taenia solium* infections using a Reed-Frost stochastic model. Int J Parasitol 37: 547–558.

75. González AE, Gilman RH, García HH, López T (2002) Use of a simulation model to evaluate control programmes against *Taenia solium* cysticercosis. In: *Taenia solium* Cysticercosis: From Basic to Clinical Science (eds. Prabhakar S, Singh G), pp. 437–448. Oxon: CAB International.

76. Torgerson PR (2006) Mathematical models for the control of cystic echinococcosis. Parasitol Int 55 (Suppl): S253–S258.

77. Roberts MG, Lawson JR, Gemmell MA (1987) Population dynamics in echinococcosis and cysticercosis: mathematical model of the life-cycles of *Taenia hydatigena* and *T. ovis*. Parasitology 94: 181–197.

78. Roberts MG (1994) Modelling of parasitic populations: cestodes. Vet Parasitol 54: 145–160.

79. WHO / FAO (2002) *Food-Borne Trematode Infections in Asia*. Joint workshop report, Ha Noi, Viet Nam, 26-28 November 2002, World Health Organization, Regional office for the Western Pacific (http://www.wpro.who.int/internet/resources.ashx/MVP/FBT.pdf)
