# Supplementary material for: A Research Agenda for Helminth Diseases of Humans: Modelling for Control and Elimination
Source: PLoS Negl Trop Dis. 2012 Apr 24;6(4):e1548. doi: 10.1371/journal.pntd.0001548 (PMC3335861; doi:10.1371/journal.pntd.0001548)
Supplement: Table S2 — Gap Analysis for Helminth Epidemiology (PDF) [file pntd.0001548.s003.pdf]

Table S2. Gap analysis for Helminth Epidemiology and Population Biology

Core theme	What we know	What research not used / applied	What not known	What research needed
Helminth epidemiology and population biology	Some parasitic helminths may have long life-spans. Life expectancy is an average duration of life, not a maximum longevity	More accurate knowledge of parasite life-span may guide minimum duration of interventions, reducing false expectations	Mean, variance, and distribution of parasite survival times (both adult worms and transmission stages)	Literature review and data gathering<br>Mathematical modelling and sensitivity / uncertainty analysis
	Parasite populations are stable, resilient, and regulated by density-dependent mechanisms	Influence of nonlinear processes on the outcome of interventions is often underestimated	Functional form, magnitude, relative contribution, and where in the parasite's life-cycle density-dependent mechanisms operate	Experimental studies where possible (e.g. vector feeding experiments in the filarial infections)<br>Fitting models to data and testing of hypotheses
	Prevalence-Intensity relationships are strongly nonlinear and determined by underlying distribution of parasites per host	Influence of overdispersed distributions is often underestimated because of focus on mean parasite load	Changes in parasite distribution among host populations resulting from intervention	Awareness of parasite distribution variance and its effects on uncertainty about infection prevalence and intensity<br>Collecting data on parasite freq. distribution as control progresses
	Age-infection profiles reflect processes of parasite acquisition-loss, and may be influenced by age-specific changes in exposure and development of immunity	Age-specific changes in the force of infection (FOI) not well quantified for helminth infections	Disentangling age from cumulative exposure effects; relative roles of exposure and immunity; immune response type, against which parasite life-stages, magnitude and duration	Quantification of FOI in sentinel (and cohort) groups as control progresses<br>Effect of co-infection interactions on age profiles of infection and morbidity
	Detection of transmission stages as indirect measures for parasitological diagnosis<br>Methods to quantify parasite presence/ abundance are inaccurate and results highly variable	Bayesian statistical methods in the absence of a gold standard infrequently used	Changes in diagnostic sensitivity as control progresses	Tools to detect parasite exposure, viability, reproductive activity in advanced stages of control / elimination<br>Assessment of infectivity to filarial vectors (xenodiagnosis)
	Exposure to parasite infection, and contamination of the environment by hosts can often vary significantly with host age (e.g. schistosomiasis)	Knowledge of infection patterns in young children will help to estimate FOI and reductions in environmental transmission with treatment	Patterns of parasite exposure and contamination in very young children (<5yrs)	Inclusion of these host sub-populations in cohort studies in M&E programmes

Table S2. Gap analysis for Helminth Epidemiology and Population Biology (Continued)

**Core theme**	**What we know**	**What research not used / applied**	**What not known**	**What research needed**
Helminth epidemiology and population biology	Laboratory-based estimates of parasite kill rates (for some helminth species)	Longitudinal studies of multiple rounds of targeted or MDA	Effectiveness of drug interventions in different field settings	Computational inference of drug effectiveness using available data
	Morbidity associated with helminth parasites is assumed to be a function of infection intensity<br><br>Multiparasitism may influence co-morbidity	Longitudinal studies of changes in morbidity and co-morbidity as a result of targeted or MDA<br><br>Studies focusing on co-infections and co-morbidities are starting	Functional relationships between parasite load and associated disease not well characterised<br><br>Are the WHO guidelines for treatment regularity been rigorously investigated given updated information on morbidity?	Statistical and mathematical methods and models for the quantitative study of parasite-induced morbidity and mortality and their temporal dynamics<br><br>Evaluation, in light of updated data, of categories to classify high, medium and low infection intensity
