# Supplementary material for: A Research Agenda for Helminth Diseases of Humans: Modelling for Control and Elimination
Source: PLoS Negl Trop Dis. 2012 Apr 24;6(4):e1548. doi: 10.1371/journal.pntd.0001548 (PMC3335861; doi:10.1371/journal.pntd.0001548)
Supplement: Table S3 — Gap Analysis for Mathematical Models (PDF) [file pntd.0001548.s004.pdf]

Table S3. Gap analysis for Mathematical Modelling of Human Helminthiases

Core theme	What we know	What research not used / applied	What not known	What research needed
Mathematical models of helminth infections	Behaviour of host-parasite systems at equilibrium	Longitudinal studies of the impact of multiple rounds of targeted or MDA on helminth infection and associated morbidity	Anthelmintic efficacy and effectiveness on various parasite life-stages and temporal changes under long-term anthelmintic treatment<br>Long-term impact of changes in parasite exposure, load, and mortality on host immune responses and reinfection rates	Models fitted to data to estimate efficacy & effectiveness of anthelmintics<br>Longitudinal immuno-epidemiological studies<br>Models fitted to cohort data to estimate changes in exposure, force of infection, treatment frequency
	Parasite and host populations are genetically diverse	Host genetics and susceptibility/predis-position to infection<br>Vector genetics and vector competence/ capacity<br>Parasite genetics regarding drug susceptibility	Molecular genetic markers of decreased drug efficacy / drug resistance starting to be developed<br>Need to distinguish genetic changes in parasite populations from programmatic issues such as low treatment coverage and compliance; reinfection from absence of clearance	Evaluation of temporal trends of treatment coverage & adherence<br>Evaluation of transmission intensity<br>Rigorous characterisation of suboptimal responses<br>Integration of parasite phenotypic and genotypic studies
	Populations of parasites, intermediate hosts and vectors are not closed entities	Studies on human population movement, flight range of vectors, transportation of snails or parasite larvae	How human movement and migration patterns affect transmission, infection and disease dynamics and re-introduction in controlled areas	Metapopulation and spatial parasite transmission models
	Morbidity control programmes aim at elimination of public health disease burden	Available data on the relationship between infection and morbidity in those programmes following longitudinal cohorts	How disease (as opposed to infection) elimination thresholds relate to burden of disease	Models for disease burden that take into account cumulative effects in addition to present infection status
	Allee effects (initial facilitation of transmission at low population densities as parasite load increases) in host-parasite systems lead to transmission breakpoints	The dynamics of transmission breakpoints scarcely studied in helminth infections of humans	Relationship between parasite breakpoints and host-parasite interactions<br>Relationship between transmission breakpoints and assumed infection thresholds in humans & vectors for elimination	Integrate models with data to explore the dynamics of transmission breakpoints for the host-parasite combinations prevailing in endemic areas<br>Use modelling to update and refine assumed elimination thresholds
	Surveillance after cessation of control is important	Mathematical models are under-utilised in surveillance systems	Relationship between model state variables and empirical surveillance tools	Epidemiological models to aid design of sampling protocols for M&E and surveillance
