# Supplementary material for: A Research Agenda for Helminth Diseases of Humans: Modelling for Control and Elimination
Source: PLoS Negl Trop Dis. 2012 Apr 24;6(4):e1548. doi: 10.1371/journal.pntd.0001548 (PMC3335861; doi:10.1371/journal.pntd.0001548)
Supplement: Text S1 — Facilitation and Limitation (PDF) [file pntd.0001548.s005.pdf]

Text S1. Facilitation and Limitation in Helminth Population Biology

The concepts of 'facilitation' and 'limitation' were originally used to describe density-dependent processes in the transmission of lymphatic filariases [1], and have been discussed in terms of filariases eradicability [2]. More generally, a parasite population process is density dependent when the rate at which the process occurs increases (positive density dependence, facilitation) or decreases (negative density dependence, limitation) with increasing parasite density.

Examples of positive (facilitating) density dependence:

i) the probability of a female worm being mated (for non-hermaphroditic parasites) within the definitive host [3], which initially increases with parasite density as there is a greater chance of worms of different sexes finding each other and mating
ii) the probability of an ingested microfilaria establishing within (anopheline and simuliid) vectors (of lymphatic filariasis and onchocerciasis) with cibarial armatures, as there is a greater chance that some microfilariae escape damage by the armature as microfilarial density increases [4]
iii) the rate of establishment of incoming parasites being facilitated (e.g. onchocerciasis [5]) by already established parasites (via immunosuppression)

Examples of negative (constraining) density dependence:

i) the limitation of microfilarial establishment and/or infective (L3) larval development within vectors of filarial nematodes as microfilarial density increases [4]
ii) the reduction in per female worm rate of egg production with increasing adult worm density [6]
iii) the reduced establishment rate of incoming worms (e.g. schistosomiasis [7]) due to established worms (via concomitant immunity)
iv) the higher mortality rate experienced by heavily infected (definitive, intermediate, or vector) hosts [4]

References

1. Pichon G, Perrault G, Laigret J (1974) Rendement parasitaire chez les vecteurs de filarioses. Bull World Health Organ 51: 517–524.
2. Duerr HP, Dietz K, Eichner M (2005) Determinants of the eradicability of filarial infections: a conceptual approach. Trends Parasitol 21: 88–96.
3. May RM (1977) Togetherness among schistosomes: its effects on the dynamics of the infection. Math Biosci 35: 301–343.
4. Basáñez MG, Churcher TS, Grillet ME (2009) *Onchocerca-Simulium* interactions and the population and evolutionary biology of *Onchocerca volvulus*. Adv Parasitol 68: 263–313.
5. Duerr HP, Dietz K, Schulz-Key H, Büttner DW, Eichner M (2003). Density-dependent parasite establishment suggests infection-associated immunosuppression as an important mechanism for parasite density regulation in onchocerciasis. Trans R Soc Trop Med Hyg 97: 242–250.
6. Walker M, Hall A, Anderson RM, Basáñez MG (2009) Density-dependent constraints on the weight of female *Ascaris lumbricoides* infections of humans and its impact on patterns of egg production. Parasit Vectors 2: 11.
7. Chan MS, Guyatt HL, Bundy DAP, Booth M, Fulford AJC, Medley GF (1995) The development of an age structured model for schistosomiasis transmission dynamics and control and its validation for *Schistosoma mansoni*. Epidemiol Infect 115: 325–344.
