# Supplementary material for: A Research Agenda for Helminth Diseases of Humans: Modelling for Control and Elimination
Source: PLoS Negl Trop Dis. 2012 Apr 24;6(4):e1548. doi: 10.1371/journal.pntd.0001548 (PMC3335861; doi:10.1371/journal.pntd.0001548)
Supplement: Text S2 — Basic and Effective Reproduction Ratios (PDF) [file pntd.0001548.s006.pdf]

Text S2. Basic and Effective Reproduction Ratios in Helminth Population Biology

The basic reproduction ratio (R_0) in helminth models is defined as the average number of female worms produced by a mature female worm during its reproductive life-span in the absence of density-dependent effects [1]. R_0 represents a somewhat idealised threshold for parasite invasion and persistence in a host population, measuring the maximum potential for parasite transmission in an infection-free state; R_0 must be greater than 1 for the parasite to invade the host population. The effective reproduction ratio (R_E) takes into account parasite density and density-dependent effects, producing a humped relationship when its (model-derived) values are plotted against worm load (Figure S2 lower panel [2,3]). This is because of the operation of initially positive (facilitating) density dependence (Allee effects), whereby increasing parasite load initially facilitates transmission (the probability of female worms being mated, or of incoming parasites establishing within the human host, or of ingested microfilariae establishing within the vector), followed by the operation of negative (limiting) density dependence (the per capita rates of parasite establishment, survival, and fecundity are constrained at high worm burdens). This results in R_E taking the value of 1 at two parasite densities; the one situated at the right of the 'hump' represents the endemic, steady state equilibrium (each female worm in the population replaces itself due to the net sum of all regulatory (negative density dependence or limitation) factors operating upon the parasite population), and the one located at the left of the hump constitutes the unstable equilibrium or breakpoint density (determined by positive density dependence or facilitation), below which the parasite population would become extinct, though it may take a long time to do so [4].

References

1. Anderson RM, May RM (1992) *Infectious Diseases of Humans. Dynamics and Control*. Oxford: Oxford University Press.
2. Churcher TS, Filipe JAN, Basáñez MG (2006) Density dependence and the control of helminth parasites. J Anim Ecol 75: 1313–1320.
3. Basáñez MG, Churcher TS, Grillet ME (2009) *Onchocerca-Simulium* interactions and the population and evolutionary biology of *Onchocerca volvulus*. Adv Parasitol 68: 263–313.
4. Gambhir M, Michael E (2008). Complex ecological dynamics and eradicability of the vector borne macroparasitic disease, lymphatic filariasis. *PLoS One* **3:** e2874.
